# Truncated titin protein in dilated cardiomyopathy incorporates into the sarcomere and transmits force

**DOI:** 10.1172/JCI170196

**Published:** 2024-01-16

**Authors:** Quentin McAfee, Matthew A. Caporizzo, Keita Uchida, Kenneth C. Bedi, Kenneth B. Margulies, Zolt Arany, Benjamin L. Prosser

**Affiliations:** 1 Cardiovascular Institute, Department of Medicine and; 2 Department of Physiology, Pennsylvania Muscle Institute, Perelman School of Medicine, University of Pennsylvania, Philadelphia, Pennsylvania, USA.

**Keywords:** Cardiology, Heart failure

**To the Editor:** Dilated cardiomyopathy (DCM) and peripartum cardiomyopathy frequently associate with heterozygous truncating variants in *TTN* (*TTN*tvs) (1). TTN codes for titin, a spring-like protein that spans the sarcomere from the Z-disk to the M-line and is essential for sarcomeric assembly, homeostasis, and regulation of contractility (2, 3).

How TTNtvs cause DCM remains unclear. Recent work by us (4) and others (5) demonstrated that the truncated titin proteins (hereafter referred to as TTNtvs) encoded by TTNtvs are expressed and detectable in human DCM hearts, concomitant with reduced full-length titin. TTNtvs have been shown in human induced pluripotent stem cell–derived (iPSC-derived) cardiomyocytes to incorporate into nascent myofibril-like structures (6). Disease may thus be caused by titin haploinsufficiency, i.e., insufficient intact titin, or by a direct negative effect of the TTNtv. With respect to the latter possibility, it is important to determine whether TTNtvs incorporate into the sarcomere in TTNtv-bearing hearts and, if so, whether they bear force. Alternatively, TTNtvs could have negative effects such as extra-sarcomeric protein aggregation (5).

To specifically detect TTNtvs in human myocardium, we developed a patient-specific TTNtv-specific antibody. One previously described DCM patient bearing a TTNtv (patient 1371) (4) had a frameshift introduced into the TTN exon 329, appending a proteome-unique sequence of 32 amino acids to the C-terminal end of the TTNtv (Figure 1A). A rabbit polyclonal antibody (FS-Ab) raised against this frameshift antigen (FS-Ag) detected the TTNtv, but not full-length titin (Figure 1B).

Probing skinned cardiomyocyte fragments from patient 1371 with this FS-Ab revealed paired stripes 238 nm ± 24 nm from the sarcomeric M-line (Figure 1C), matching the predicted location of the TTNtv C-terminus along the thick filament, assuming integration matching that of full-length titin. We conclude that this TTNtv integrated into the sarcomere at the expected thick filament binding location and did so despite not binding to the M-line.

We next investigated whether TTNtvs can bear the length-dependent forces generated by the entropic spring behavior of titin’s I-band. Upon stretch of cardiomyocyte fragments from very short to supraphysiologically long sarcomere lengths (SLs), TTNtv from patient 1371 remained attached to the relatively inelastic thick filament (Figure 1D). The distance between TTNtv C-termini (i.e., across the M-line) remained invariant, whereas the distance to the Z-disk (i.e., spanning the I-band) increased with SL (Figure 1D quantification). Labeling the N-terminus of titin (titin-Z, in blue in Figure 1) revealed no signal peaks outside the Z-disk, demonstrating that the N-terminus of the TTNtv remained attached to the Z-disk. We conclude that TTNtvs remained attached to both the Z-disk and thick filament upon stretch, indicating that TTNtv can bear load across the sarcomere.

To test in a different manner whether TTNtvs bind to the Z-disk, we stretched cardiomyocyte fragments from patient 1371 and added 400 mM KCl, a treatment known to disrupt the thick filament. We reasoned that release of TTNtv from the thick filament would enable entropic spring forces to pull its truncated C-terminus toward the Z-disk. Under high-salt treatment, the TTNtv C-terminus signal relocated from the thick filament to near the Z-disk (Figure 1E, Supplemental Figure 1, and Supplemental Methods; supplemental material available online with this article; https://doi.org/10.1172/JCI170196DS1), although, interestingly, not precisely to the Z-disk, consistent with previous observations that titin binds the thin filament outside the Z-disk (3). In contrast, full-length titin remained attached to the M-line (Supplemental Figure 2). These data confirm that TTNtvs transmitted force across the I-band region of the sarcomere, even at supraphysiological SLs, but detached more readily than full-length titin from the thick filament. The observed recoil of the truncated C-terminus to the Z-disk also demonstrates that the recognized protein was not encoded by the Cronos transcript, as the latter lacked the I-band spring and the Z-disk attachment regions. The data also demonstrate that attachment of titin to the M-line was dispensable for binding to the Z-disk and incorporation into the sarcomere.

Together, these observations demonstrate that TTNtv protein was present in myocardium from a TTNtv-bearing DCM patient; that the TTNtv protein was incorporated into the sarcomere akin to full-length titin, binding both the Z-disk and the thick filament; and that this A-band overlapping truncated titin bore force across the I-band, even under supraphysiological strain, but detached more readily from the thick filament compared with full-length titin (Figure 1F).

Caveats of our study include the restriction to a single patient; we were not able to generate antibodies against other TTNtv frameshift variants. The data also do not provide stoichiometric information about truncated versus full-length titin protein.

While not ruling out contributions of haploinsufficiency or of aberrant protein aggregation, our study supports the notion that truncated titin proteins may have a direct effect on sarcomeric behavior, affecting sarcomeric structure, contractility, or signaling, and thus contributing to TTNtvs-associated DCM pathophysiology.

## Supplementary Material

Supplemental data

Unedited blot and gel images

Supporting data values

## Figures and Tables

**Figure 1:**
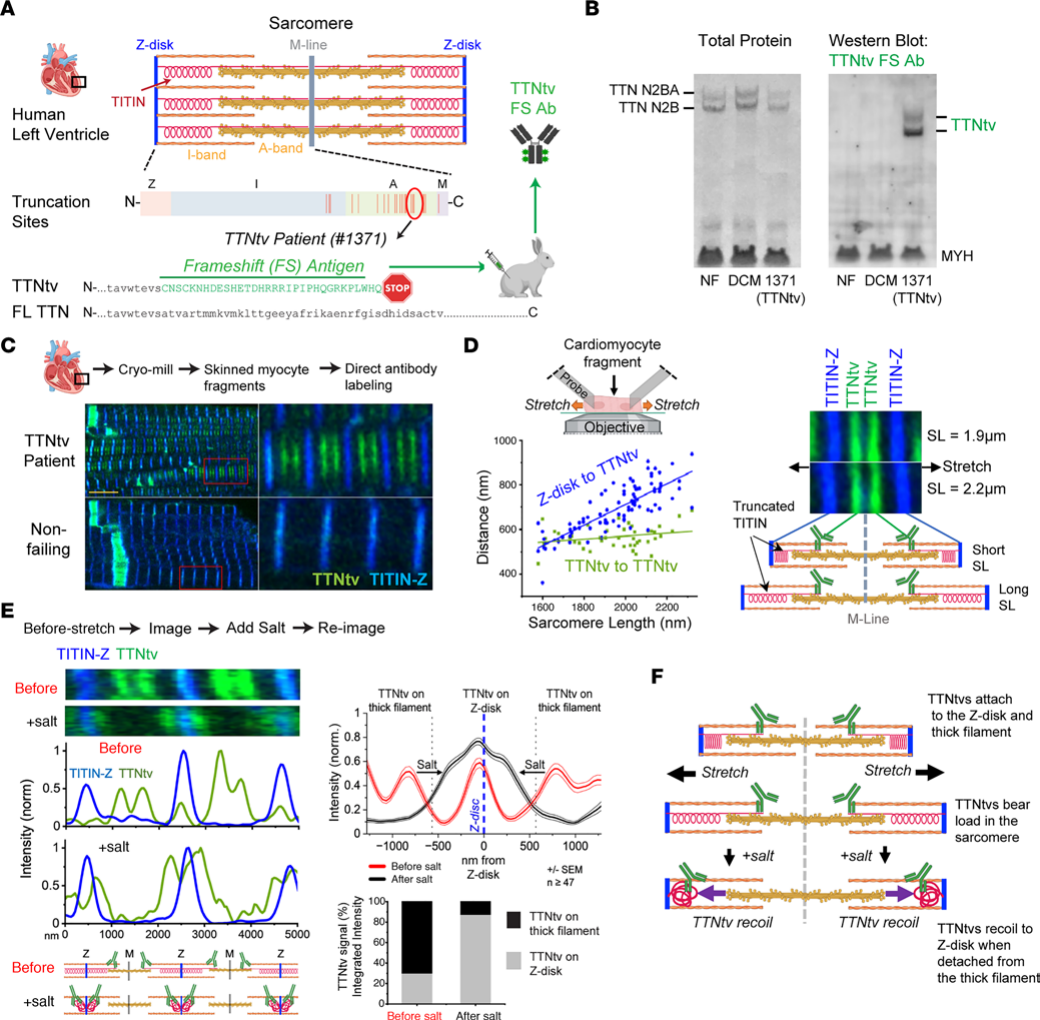
Truncated titin is present in the myocardial sarcomere and transmits force from the Z-disk to the thick filament. (**A**) Schematic showing titin frameshift truncation and the associated C-terminal FS-Ag against which a specific FS-Ab has been made. (**B**) Western blot showing that the FS-Ab detects truncated titin and not full-length titin. NF, nonfailing. (**C**) Human cryo-crushed cardiomyocyte fragments from patient 1371 and a control heart labeled with truncated titin FS-Ab in green and anti–Z-disk titin in blue. (**D**) Quantification and images of fragments from patient 1371 stained as above and stretched. (**E**) Cardiomyocyte fragments from patient 1371, labeled as above, that were stretched, imaged, treated with 400 mM KCl, and re-imaged. Quantifications are from 47 or more sarcomeres per state, ± SEM. (**F**) Schematic showing that truncated titin bears load across the sarcomere and is attached to both the Z-disk and thick filament.

## Version 1 11/09/2023

In-Press Preview

## Version 2 01/16/2024

Electronic publication
