# Supplementary material for: Truncated titin protein in dilated cardiomyopathy incorporates into the sarcomere and transmits force
Source: J Clin Invest. 2024 Jan 16;134(2):e170196. doi: 10.1172/JCI170196 (PMC10786684; doi:10.1172/JCI170196)
Supplement: Supplemental data [file jci-134-170196-s024.pdf]

Supplemental Figures and Methods:

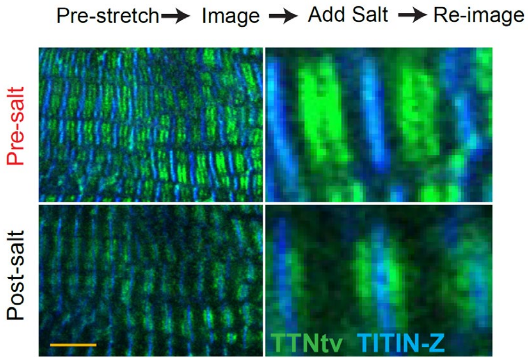

Supplemental figure 1: Truncated titin retracts to the Z-disk after 400mM KCl treatment. Patient 1371 fragment stained as in Figure panel E. The green channel is truncated titin FS-Ab, and the blue channel is the Z-disc (N-terminus) of both full length and truncated titin. Scale bar is 5μM.

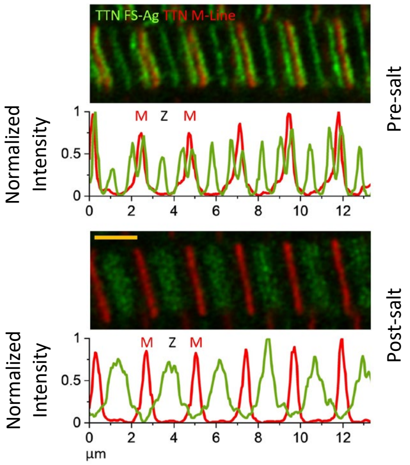

Supplemental figure 2**:** Full length titin remains attached to the M-line under high salt conditions. Patient 1371 cardiomyocyte fragment stained for FS-Ab and M-line full length titin and treated as in Figure Panel E. The green channel is truncated titin FS-Ab, the red channel is the C-terminus of full-length titin. Scale bar is 2μm. Plots represent normalized signal intensity profiles along the length of myofibril.

Supplemental Materials and Methods: Human LV tissues were acquired under protocols and ethical regulations approved by institutional Review Boards of the University of Pennsylvania and the Gift-of-Life Donor Program (Pa, USA) as previously described in[1,2]. Patient 1371 anti truncated titin frameshift antigen antibody was generated against the frameshift antigen sequence using standard rabbit polyclonal antibody generation methods by ThermoFisher Scientific custom antibody production services. Non-patient specific antibodies (Anti-titin N-Terminus, TTN-1; and anti titin C-terminus, TTN-9) were supplied by Myomedix of Neckargemuend, Germany.) Titin protein extraction and immunoblots were performed as previously described[2]. For imaging studies, frozen tissue was pulverized on liquid nitrogen into cardiomyocyte fragments and stored at -80xxx. Aliquots of this powder were suspended in relaxing buffer (10mM MOPS, 2mM EGTA, 8mM 2NaATP, 5mM $MgCl_2$, 5mM BDM, with 2x concentration Roche Mini protease inhibitor tablets, set to pH 7.0 with KCl.) with Triton X-100 for 10-20 minutes then exchanged into relaxing buffer lacking triton and stained with directly labeled antibodies for approximately 20 minutes before imaging. Samples were transferred to a coverslip and superfused with relaxing buffer on a Zeiss 880 Airyscan equipped microscope also equipped with a Myostretcher with apparatus from IonOptix. Cardiomyocytes or cardiomyocyte fragments were immobilized on the coverslip with custom rigid probes for imaging and stretching. For truncated titin recoil studies, superfusing relaxing buffer was replaced with relaxing buffer supplemented with 400mM KCl before re-imaging.[3] For data found in figures see Excel file containing the Supporting Data Values.

Supplemental references:

1. Chen CY, Caporizzo MA, Bedi K, et al. Suppression of detyrosinated microtubules improves cardiomyocyte function in human heart failure. *Nature Medicine*. 2018;24(8):1225-1233. doi:10.1038/s41591-018-0046-2
2. McAfee Q, Chen CY, Yang Y, et al. Truncated titin proteins in dilated cardiomyopathy. *Sci Transl Med*. Nov 3 2021;13(618):eabd7287. doi:10.1126/scitranslmed.abd7287
3. Tawada K, Yoshida A, Morita K. Myosin-free ghosts of single fibers and an attempt to re-form myosin filaments in the ghost fibers. *J Biochem*. Jul 1976;80(1):121-7. doi:10.1093/oxfordjournals.jbchem.a131243
