# Supplementary material for: Truncated titin protein in dilated cardiomyopathy incorporates into the sarcomere and transmits force
Source: J Clin Invest. 2024 Jan 16;134(2):e170196. doi: 10.1172/JCI170196 (PMC10786684; doi:10.1172/JCI170196)
Supplement: Unedited blot and gel images [file jci-134-170196-s025.pdf]

Unedited Gel and Blot images:

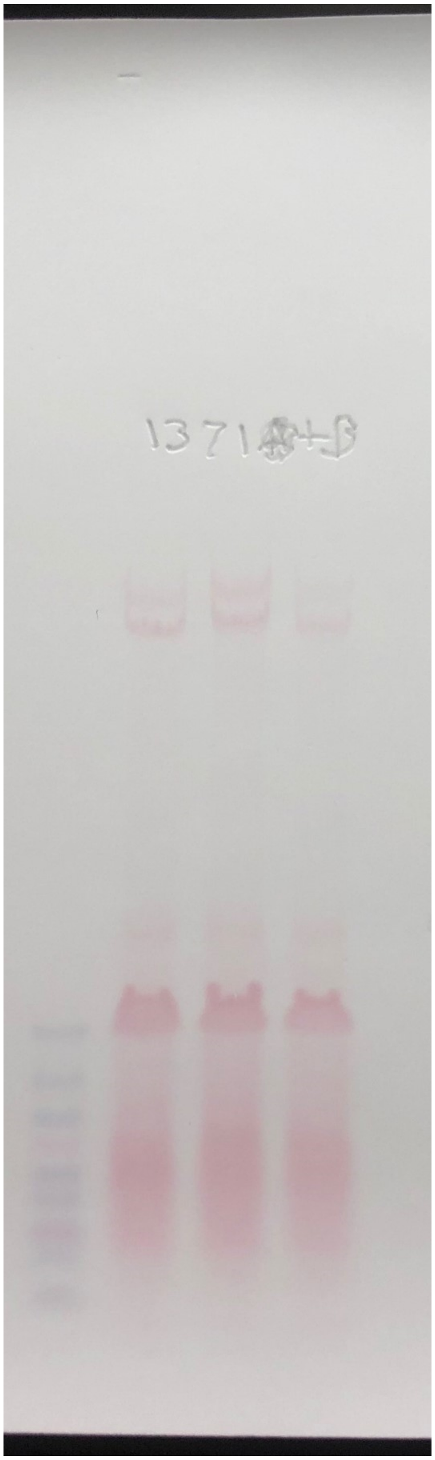

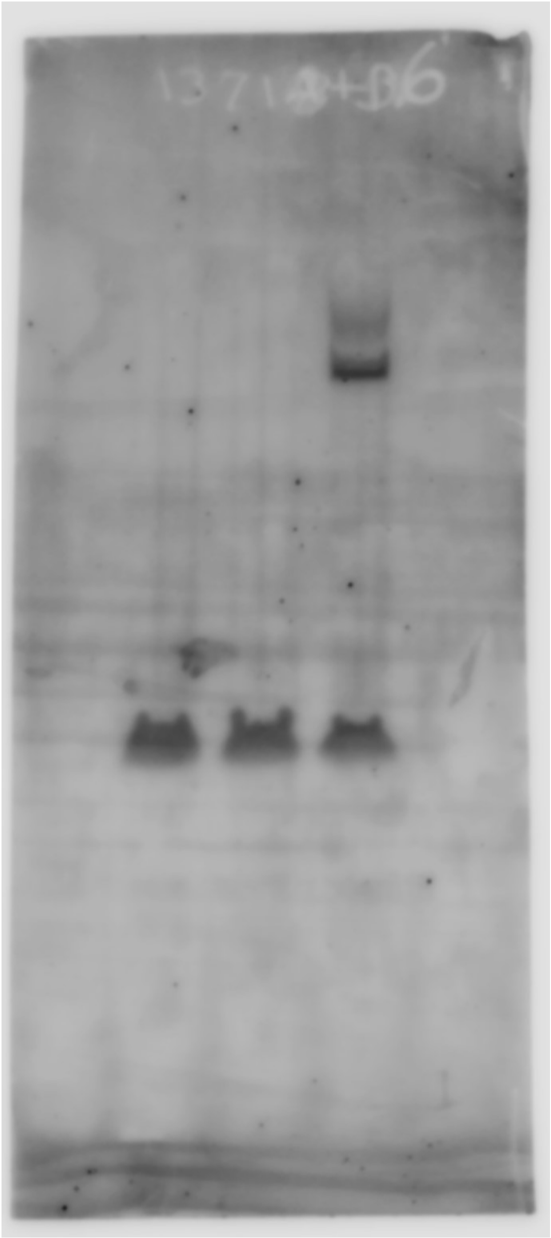

Unedited blots related to Figure Panel 2: Left, Ponceau stain imaged with color camera. Right, chemiluminescent western blot.
